# Opioid system and related ligands: from the past to future perspectives

**DOI:** 10.1186/s44158-024-00201-2

**Published:** 2024-10-11

**Authors:** Laura Rullo, Camilla Morosini, Antonio Lacorte, Marco Cristani, Flaminia Coluzzi, Sanzio Candeletti, Patrizia Romualdi

**Affiliations:** 1 Department of Pharmacy and Biotechnology, Alma Mater Studiorum — University of Bologna, Irnerio 48, Bologna, 40126 Italy; 2 Department of Medical-Surgical Sciences and Translational Medicine, Sapienza University of Rome, Rome, Italy; 3 Unit of Anaesthesia, Intensive Care and Pain Medicine, Sant’Andrea University Hospital, Rome, Italy

**Keywords:** Opioids, Chronic pain, Bifunctional agonists, Biased agonists, Tolerance, OUD (opioid use disorder), OIH

## Abstract

Chronic pain is a pathological condition affecting about 30% of population. It represents a relevant social-health issue worldwide, and it is considered a significant source of human suffering and disability, strongly affecting patients’ quality of life. Despite several pharmacological strategies to guarantee an adequate pain management have been proposed over the years, opioids still represent one of the primary choices for treating moderate-to-severe pain in both cancer and non-cancer patients. However, chronic use of opioids often leads to numerous side effects, including respiratory depression, constipation, analgesic tolerance, and opioid-induced hyperalgesia (OIH), which can strongly limit their use. Given the fundamental role of opioid system in pain relief, this review provides a general overview about the main actors (endogenous opioid peptides and receptors) involved in its modulation. Furthermore, this review explores the action and the limitations of conventional clinically used opioids and describes the efficacy and safety profile of some promising analgesic compounds. A deeper understanding of the molecular mechanisms behind both analgesic effects and adverse events could advance knowledge in this field, thus improving chronic pain treatment.

## Background

Chronic pain, defined as any pain persisting beyond 3 months, represents a relevant social-health issue [1]. It is estimated that approximately 30% of the general population suffers from this pathological condition [2, 3]. Although mortality rates are higher for other diseases, chronic pain represents a significant source of human suffering and disability, severely affecting patients’ quality of life and psychological well-being [3].

Despite numerous strategies for effective chronic pain management have been proposed in recent years, opioids remain a primary choice for treating moderate-to-severe pain in both cancer and non-cancer patients [4, 5]. However, their prolonged use is frequently hindered by a wide range of side effects, including respiratory depression, constipation, nausea, and itching [2, 6]. Additionally, chronic opioid administration often leads to a paradoxical increase in pain sensitivity (opioid-induced hyperalgesia, OIH) and molecular adaptive changes, which contribute to a drug’s efficacy reduction over time (i.e., tolerance) and physical dependence [7, 8]. Moreover, the outspread of opioids crisis in the USA, mainly driven by increased abuse, misuse, and diversion, has strongly affected opioid prescriptions also in other countries [9, 10].

Therefore, in the last years, numerous efforts were made by scientific community in the attempt to identify innovative analgesics characterized by similar potency and efficacy compared to the common opioid agonists (i.e., morphine, oxycodone, fentanyl) but with a better tolerability profile (fewer side effects and abuse liability). In this frame, a lot of pharmacological approaches (mixed pharmacology, functional selectivity) has been proposed to develop new analgesic drugs [11–16]. On these bases and given the relevance of endogenous opioid system in pain management, this review examines the beneficial effects of both traditional and novel compounds targeting opioid receptors, aiming to enhance understanding in this field and thus improving the chronic pain treatment.

### The endogenous opioid system

The endogenous opioid system is a complex network of neurons and receptors that is distributed either in the central nervous system (CNS) and in the periphery, where it modulates different functions, from the well-known nociceptive transmission to the regulation of gastrointestinal, endocrine, and autonomic functions, as well as reward mechanisms and mood and stress-response processes [17].

This endogenous system represents the target of the pharmacological actions of opiates, originally derived from the extract of the juice of the poppy plant capsule (*Papaver somniferum*). The term opiate is used to describe the naturally occurring alkaloid morphine and its synthetic derivatives, which represents the prototype for narcotic analgesics, and should not be confused with the term opioid, preferably. In fact, while the term opioids refer to neuromodulators that are the physiological ligands of opioid receptors [18], the word opiates refers to xenobiotic drugs/molecules capable to mimic the actions of the endogenous opioid peptides at the level of the central and peripheral nervous systems. However, currently and for some time, doctors prefer to use the term opioids for both [17].

### Endogenous opioid peptides

The endogenous opioid system is characterized by distinct families of opioid neuropeptides, *β*-endorphins, enkephalins, and dynorphins, respectively, which represent the endogenous ligands for the different classic three opioid receptors. Each of these peptides derives from the proteolytic cleavage of three distinct polypeptide precursors: proopiomelanocortin (POMC), proenkephalin (PENK), and prodynorphin (PDYN), coded by their corresponding genes (*POMC*, *PENK*, and *PDYN*) [19].

These endogenous opioid ligands exhibit different affinities for each opioid receptor, but all of them share a common amino N-terminal tetrapeptide sequence (Tyr-Gly-Gly-Phe) that allows the interaction with the receptors (Table 1) [20].

**Table 1:** Endogenous mammalian opioid peptides, amino acid sequence, and selectivity for the opioid receptors

Endogenous opioid peptide	Amino acid sequence (three-letter code)	Opioid receptor affinity
*β*-endorphin	**Tyr**-**Gly**-**Gly**-**Phe**-Met-Thr-Ser-Glu-Lys-Ser-Gln-Thr-Pro-Leu-Val-Thr-Leu-Phe-Lys-Asn- Ala-Ile-Ile-Lys-Asn-Ala-Tyr-Lys-Lys-Gly-Glu	MOR, DOR (MOR = DOR)
Met-enkephalin	**Tyr**-**Gly**-**Gly**-**Phe**-Met	DOR, MOR (DOR > > MOR)
Leu-enkephalin	**Tyr**-**Gly**-**Gly**-**Phe**-Leu	
Dynorphin A	**Tyr**-**Gly**-**Gly**-**Phe**-Leu-Arg-Arg-Ile-Arg-Pro-Lys-Leu-Lys-Trp-Asp-Asn-Gln	KOR, MOR, DOR
Dynorphin B	**Tyr**-**Gly**-**Gly**-**Phe**-Leu-Arg-Arg-Gln-Phe-Lys-Val-Val-Thr	KOR > > MOR and DOR
Nociceptin/orphanin FQ	Phe-Gly-Gly-Phe-Thr-Gly-Ala-Arg-Lys-Ser-Ala-Arg-Lys-Leu-Ala-Asn-Gln	NOP

In addition to these, a fourth gene (*PNOC*) has been later discovered. This gene codes for pronociceptin (PNOC), which is the precursor of the peptide named nociceptin that was initially also called as nociceptin/orphanin FQ (F stands for phenylalanine and Q for glutamine, which are its first and last amino acids) [19]. This neuropeptide lacks the N-terminal tetrapeptide sequence, and although there are some sequence similarities between PNOC and PDYN, this peptide has minimal affinity for the opioid receptors. These are the reasons why nociceptin acts via a different type of receptor, the opioid receptor-like receptor (formerly known as ORL1) now called nociceptin opioid receptor (NOP), and together with it represents the fourth family of the entire endogenous opioid system. This last family is characterized by partially different properties and can also functionally antagonize the classical opioid system [21].

**Fig. 1:**
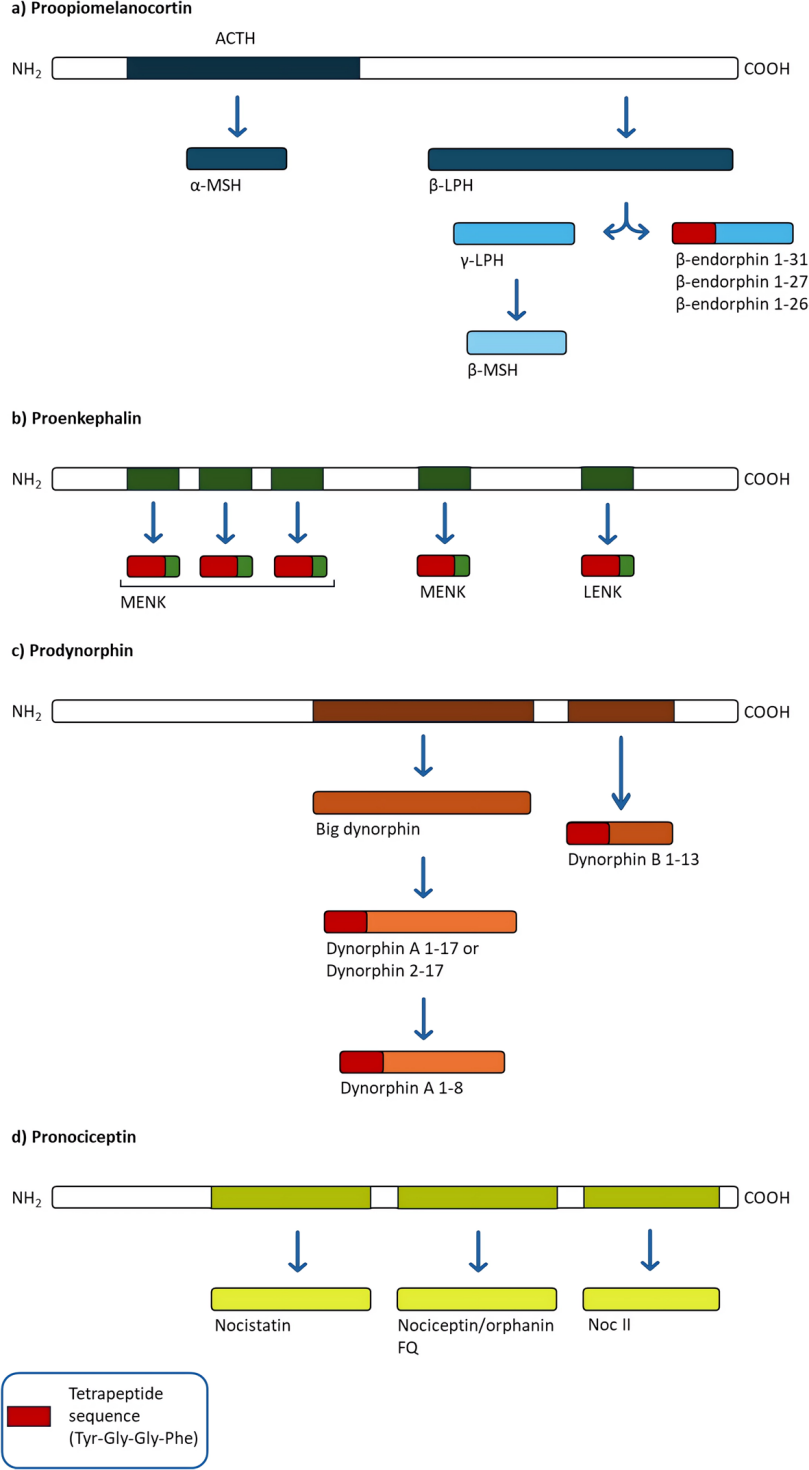
Opioid peptide precursors. Representative peptides derived from the precursors proopiomelanocortin (**a**), proenkephalin (**b**), prodynorphin (**c**), and pronociceptin (**d**)

The processing of the precursors is a complex metabolic procedure that affects the resulting bioactivity of the peptides (Fig. 1). It begins in the endoplasmic reticulum, but the majority of the processing occurs in the secretory vesicles, where the precursors and their processing enzymes have been packaged [22, 23].

*POMC* gene encodes for a lot of peptides, either opioid and non-opioid, and the polypeptide POMC is the only known precursor for the opioid neuropeptide *β*-endorphin. POMC is first cleaved into two peptides: adrenocorticotropic hormone (ACTH), which can also be later hydrolyzed to *α*-MSH, at the N-terminal ending, and to *β*-lipotropin (*β*-LPH) at the C-terminal ending. Subsequently, *β*-LPH is further processed to generate the full-length *β*-endorphin 1–31, *β*-MSH, and *γ*-lipotropin (*γ*-LPH) (Fig. 1a). It is interesting to note that the presence of ACTH and *α*-MSH as product of POMC processing could suggest the existence of neuroendocrine correlations between the opioid system and responses to stress conditions (hypothalamic–pituitary–adrenal axis).

PENK generates six copies of met-enkephalin (MENK), four of which are expected to be cleaved and one copy of leu-enkephalin (LENK) [24], whereas the dynorphin family peptides, including dynorphin 1–8, dynorphin A 1–17, dynorphin B 1–13, and *α*- and *β*-neo-endorphin, all stem from the precursor PDYN (Fig. 1b, c).

As previously reported, PNOC is the precursor of nociceptin and is processed to form three main peptides: nociceptin, nocistatin, and NocII/NocIII. Nowadays, the nocistatin and NocII/NocIII target receptors are unknown (Fig. 1d) [25].

Not only these well-characterized peptides but also other opioid receptor-interacting peptides have been identified, namely deltorphins and dermorphins, isolated from amphibian skin, and endomorphins, isolated from rodent brains and binding with high affinity and selectivity to the *μ*-opioid receptor.

### Opioid system distribution

The endogenous opioid pathways have a specific organization in the CNS [17, 26, 27]. Neurons synthesizing POMC and, thereby, *β*-endorphin are localized in the arcuate nucleus of the hypothalamus and in the solitary tract nucleus in the dorsal medulla. In turn, neurons of the arcuate nucleus tract project to limbic forebrain and midbrain areas, including nucleus accumbens (NAc), amygdala, hypothalamus, periaqueductal gray (PAG), and ventral tegmental area (VTA), whereas neurons of the solitary tract nucleus project mainly to brainstem and spinal cord. In addition to CNS, *β*-endorphin is also produced in some peripheral tissues such as placenta, pancreas, testis, gastric antrum mucosa, and adrenal medulla.

In contrast, enkephalins are widely distributed throughout the CNS. Indeed, PENK is abundantly expressed in areas involved in the modulation of nociceptive transmission (laminae I and II of the spinal cord, spinal trigeminal nucleus, PAG matter), in the control of motor activity (substantia nigra, caudate) and memory/affective behavior (NAc, hippocampus, amygdala, locus coeruleus, anterior olfactory nucleus, cerebral cortex), in neuroendocrine functions (hypothalamus), and in the regulation of the autonomic nervous system (medulla oblongata).

Dynorphins have a distribution similar to enkephalins in the CNS; they are present in lamina II of the spinal cord and in the anterior hypothalamic nucleus whose axons project to the posterior hypophysis, caudate, reticular formation, hippocampus, and cerebral cortex. Finally, nociceptin is present in the CNS and peripheral tissues.

The endomorphins have been identified by immunochemistry in the outer layers of the spinal cord dorsal horns, nucleus ambiguous, spinal trigeminal nucleus, NAc, thalamic nuclei, septum, hypothalamus, amygdala, locus coeruleus, and PAG.

### Opioid receptors and signaling transduction

Opioid receptors are extensively distributed across the central and peripheral nervous systems. Their predominant presence in pain-modulating descending pathways underscores their critical role in analgesia. However, numerous studies have emphasized their involvement in a wide range of behavioral effects, including stress responses, depression, anxiety, reward/aversion behaviors, gastrointestinal transit, and neuroendocrine and immune functions [28]. The endogenous opioid system consists of four seven-transmembrane G protein-coupled receptors (GCPRs), specifically *µ* (MOR), *κ* (KOR), *δ* (DOR), and opioid receptor-like 1 (NOP). After activation, these opioid receptors interact with inhibitory G proteins (Gαi and Gαo), resulting in cell hyperpolarization and in a decrease in neurotransmitter release. When an endogenous or exogenous opioid agonist binds to the extracellular N-terminal domain of the receptor, the Gαi/o protein on the intracellular C-terminal side binds to GTP (guanosine triphosphate). This binding causes the Gαi/o protein dissociation from Gβγ subunits and the subsequent modulation of downstream intracellular signaling cascade. In particular, opioid receptors stimulation has been shown to inhibit adenylate cyclase (AC) activity, thereby preventing the production of cyclic AMP (cAMP) [29, 30].

Moreover, these receptors are able to modulate calcium and potassium ion channels in order to reduce neuronal excitability and transmitter release. Specifically, the Gα subunit can directly interact with the G protein-gated inwardly rectifying potassium channel (GIRK), Kir3, resulting in cellular hyperpolarization and the inhibition of tonic neural activity [31, 32] while the directly binding of the dissociated Gβγ subunit to calcium channels inhibits calcium conductance by reducing voltage activation of channel pore opening [33].

**Fig. 2:**
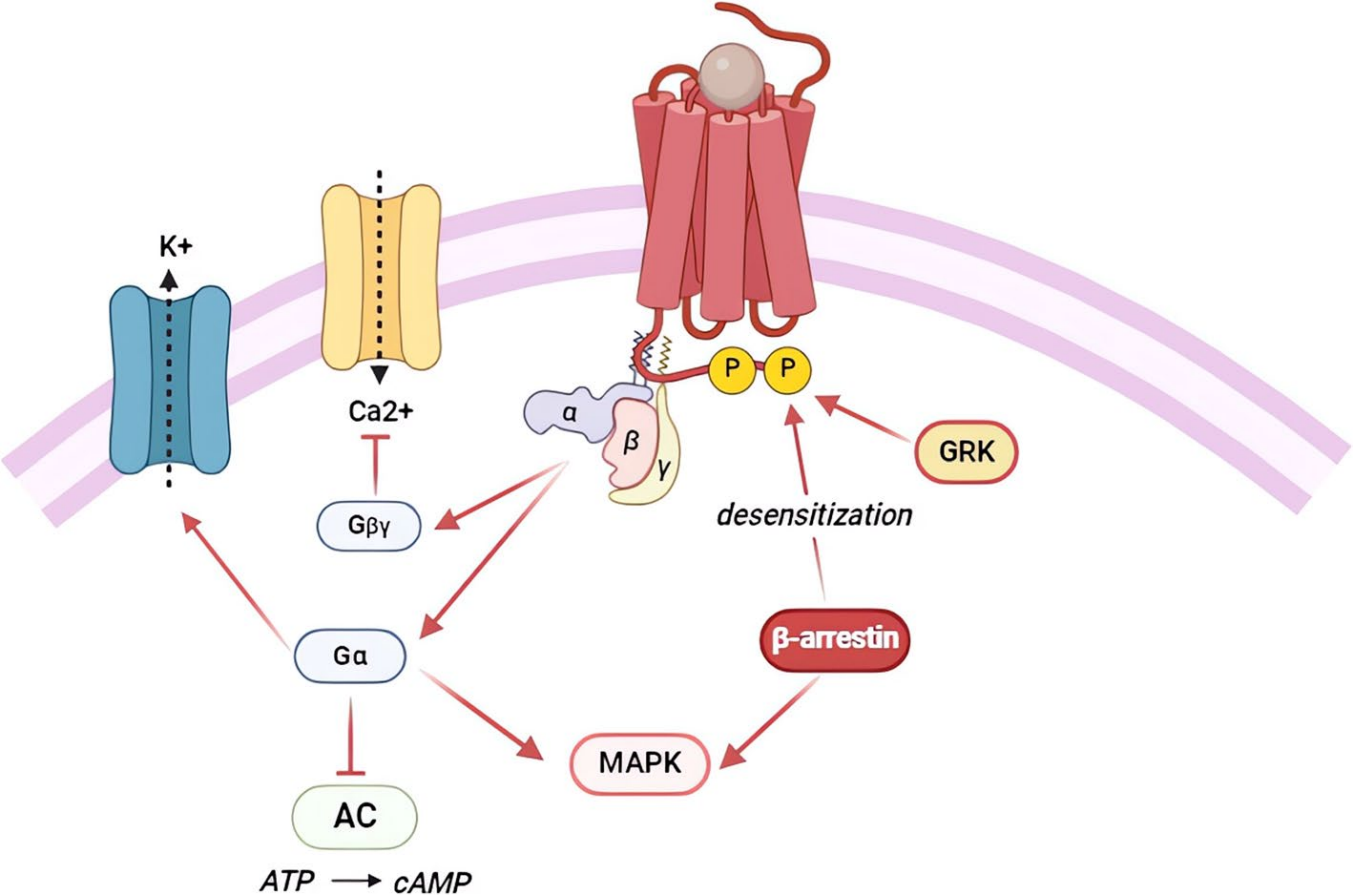
Intracellular opioid receptor signaling. Ligand activation of opioid receptors causes the dissociation of the Gαi/o protein from the Gβγ subunit, subsequently modulating downstream intracellular signaling cascades. The Gα subunit mediates the activation of K^+^ channels and the inhibition of adenylate cyclase, resulting in cell hyperpolarization and decreased intracellular cAMP levels. Meanwhile, the Gβγ heterodimers inhibit voltage-gated calcium channels, thereby reducing calcium influx. Sustained activation of the G protein can lead to phosphorylation of the opioid receptors by G protein-coupled receptor kinases, resulting in the recruitment of *β*-arrestin protein, thus facilitating receptor internalization

In addition, opioid receptor stimulation has been shown to produce phospholipase C (PLC) and mitogen-activated protein kinase (MAPK) activation [29] (Fig. 2).

Upon G protein-sustained activation, opioid receptors may be subject to regulation by G protein signaling (RGS) proteins or desensitization. Generally, the desensitization process is promoted through the receptors phosphorylation by G protein-coupled receptor kinases (GRKs) or other second messenger-regulated kinases such as protein kinase C (PKC). After that, arrestin proteins are recruited in order to block further G protein coupling and to facilitate receptor internalization via association with clathrin and clathrin-adaptor proteins [29] (Fig. 2). Following internalization, receptors may either be recycled back to the plasma membrane or degraded depending on which specific residues are phosphorylated [34].

### Tolerance and physical dependence to opiates

Chronic exposure to opioids leads to the development of two closely related pharmacological phenomena: tolerance and physical dependence. Tolerance is characterized by a diminished response to the drug over time that occurs after repeated or prolonged use. As a result, higher doses of opioids are often required to achieve the same level of pain relief. This escalation in dosage disrupts the body’s homeostasis, ultimately resulting in physical dependence. Physical dependence manifests as withdrawal symptoms when opioid use is abruptly discontinued. Notably, these phenomena can be viewed as independent of psychic dependence.

Tolerance results from a series of cellular adaptation processes, including desensitization and receptor downregulation, which lead to a reduction in the number of functional receptors present on the cell membrane. Tolerance can be classified as either acute or chronic, depending on the duration of receptor exposure to agonists. Like other receptors of GPCR family, agonist binding to opioid receptors activates a transduction signal pathway leading to short-term events of desensitization that as abovementioned involve receptor phosphorylation by kinases and that may lead to tolerance. Following desensitization, the internalization process occurs via endocytosis [29, 35]. Receptor internalization is mediated by GRKs that phosphorylating the agonist-bound receptor promotes its interaction with *β*-arrestin. From a molecular perspective, chronic tolerance has been related to the superactivation of cAMP pathway and to adaptations of the G protein subunits αi/o and βγ complex [35, 36].

The increase in cAMP has also been suggested as a mechanism responsible for the development of physical dependence. In fact, this metabolite has been reported to be upregulated in several brain nuclei. In the nucleus accumbens, the upregulation of the cAMP-PKA pathway promotes an increase in CREB-mediated prodynorphin transcription [35]. This results in significant stimulation of presynaptic KOR and the subsequent inhibition of dopamine release, contributing to the characteristic dysphoria of opioid withdrawal syndrome. In addition, the activation of the cAMP-PKA-CREB signaling cascade in the locus coeruleus participates to the manifestation of dysphoria and somatic symptoms associated to this phenomenon [37, 38].

Furthermore, it has been reported that opioids treatment induces gene expression alteration of several proteins such as neurotransmitters, mediators, and transcription factors, known to be involved in the long-term molecular and cellular adaptation occurring during tolerance and dependence [39].

### Opioid use disorder

Opioid use disorder (OUD) is characterized by the loss of control over the intake and seeking of drugs of abuse, a disorder that is called compulsive behavior. This represents a chronic and recurrent disorder with serious negative consequences to self or others [40].

The mesocorticolimbic system is the neural circuit responsible of the reinforcing effects of all drugs of abuse. It is mainly made up of dopaminergic neurons, and the ventral tegmental area represents their source. In fact, dopaminergic neurons project from VTA to the nucleus accumbens via mesolimbic pathway and to the prefrontal cortex (PFC) via mesocortical pathway.

Not only the dopaminergic but also glutamatergic and GABAergic neurons are involved in this circuit. Indeed, they project from the PFC and amygdala to the NAc and from the NAc to the VTA, respectively [41, 42].

Opioid interneurons are present in the NAc, amygdala, and VTA. The opiates but also the endogenous opioids enhance the release of dopamine directly activating the *μ* and *δ* receptors in the NAc and indirectly the *μ* receptors on GABAergic neurons of the VTA. In the VTA, the inhibition of GABAergic neurotransmission is responsible for the increasing activity of dopaminergic neurons [42, 43].

In contrast, the activation of *κ* receptors, localized on the cell bodies of the dopaminergic neurons in the VTA and on the terminations in the NAc, inhibits the dopaminergic transmission in the mesocorticolimbic circuit [44].

Three factors that may contribute to the vulnerability of developing an addiction has been proposed: factors related to the substance effects and environmental and genetic factors (polymorphisms in the chromosomes containing the genes coding for the opioid system) [45, 46].

Currently, the incorrect idea that the use of opiates will inevitably lead to the psychic dependence limits their therapeutic use for the treatment of chronic pain. In fact, the therapeutic use of opiates does not associate environmental conditioning that represents a very important element in establishing the positive reinforcement that leads to the compulsive use. Indeed, it has been reported that pain induced specific molecular adaptation (e.g. PKC and ERK) which results in a decrease in the reward effect of exogenous MOR agonists [47]. Therefore, the condition in which drug is taken and the molecular changes occurring during chronic pain typically do not represent a favorable environment for developing AUD. In this context, clinical findings support and confirm that OUD is very uncommon in patients suffering from chronic pain. Furthermore, preclinical data indicate that the neuropathic pain condition causes the release of the opioid peptide *β*-endorphin in the VTA, so inducing desensitization of the MOR controlling the activity of dopaminergic neurons and the DA release in the NAc. The result is a lack of DA in the area devoted to the rewarding effects; in addition, a role is played together with a role exhibited by BDNF-releasing glial cells that inverts the GABA A receptors function and blocks reward [47–49].

### Opioid agonists

#### Morphine

Morphine was isolated from opium for the first time in 1806 by Serturner that called it with this name from god of sleep Morpheus because of its apparently hypnotic properties [50]. Morphine is one of the most widely used drugs for the treatment of acute or severe chronic pain. Like other drugs in this class, it shows affinity for MOR, DOR, and KOR. However, it principally exerts its analgesic effects binding to MOR in both CNS and the peripheral nervous system (PNS) [51, 52]. In particular, morphine promotes a reduction of nociceptive transmission between the first- and the second-order neurons and by activating the descending inhibitory pathway at CNS level as well as probably inhibiting the nociceptive afferent neurons of the PNS [17, 51, 53]. The use of morphine is associated with common side effects including severe constipation, respiratory depression, itching, nausea, vomiting, and urinary retention. Furthermore, its prolonged use is often limited by the rapid development of analgesic tolerance, OIH and, physical dependence [51, 54]. Morphine is the progenitor of the family of compounds called opiates and through chemical modifications of its structure, and other compounds such as oxycodone and heroin have been synthesized**.**

#### Oxycodone

Oxycodone is a semisynthetic opiate derived from thebaine (an alkaloid naturally occurring in opium juice together with morphine but pharmacologically inactive per se). It has been introduced on the pharmaceutical market in 1939. It is a strong opioid, usually used as an alternative to morphine. It acts as a selective MOR agonist. However, it shows less binding affinity for MOR than morphine or methadone [55, 56]. Oxycodone is generally used in patients experiencing moderate-to-severe cancer pain as well as for the treatment of a wide range of severe nonmalignant-related pain conditions [57]. Despite oxycodone demonstrated to be very effective in providing an adequate pain relief, its use has been often restricted over the years due to the development of typical opioid side effects including bowel dysfunction. However, the introduction of formulations that combine prolonged-release oxycodone and naloxone has been shown to provide effective pain relief while reducing the risk of opioid-induced constipation [57]. Oral naloxone undergoes a relevant liver first-pass metabolism, which reduces to about 2% the amount of drug that crosses the blood brain barrier and may interfere with analgesia [58].

Oxycodone is one of the opiates behind the serious opioid crisis that broke out in the United States (US) between the late 1990s and the 2000s. Indeed, the inappropriate prescription of this medication and high abuse liability of this drug have led to an increase of overdose rates deaths [59].

#### Fentanyl and its analogues

Fentanyl was synthesized by Janssen Pharmaceutica in 1960 in order to produce a new opioid analgesic with enhanced analgesic potency and fewer adverse effects compared with morphine [60]. Indeed, fentanyl is a full *μ*-opioid receptor agonist, 70–100 times more powerful than morphine, and due to its features, it is applied as an adjuvant in anesthesia, for sedation and the treatment of acute and chronic pain [61, 62]. It induces similar morphine side effects. However, it produces less cardiovascular effects than morphine [17].

In the recent years, a growing concern arose about fentanyl and its analogues, widely synthesized in illicit laboratories, and adulterated with other illicit drugs such as heroin, which could contribute to the exponential growth in the number of drug-related overdose deaths. In fact, the rapid death derived from fentanyl administration has become increasingly more common [63]. Its high potency and fast onset of action may explain a high risk of overdose deaths, often occurring for severe respiratory depression [63, 64].

Among its derivates, sufentanil, remifentanil, and alfentanil are the most commonly used in clinical practice. They have an analgesic potency similar to fentanyl, but they differ from each other for chemical substitutions on piperidine ring that change the pharmacokinetics characteristics (i.e., bioavailability, lipid solubility, binding to plasma proteins) [62]. Their prescription allows for their use as potent analgesics in severe acute pain therapy (intraoperative and postoperative analgesia), even by infusion, but only in specific conditions, such as in monitored patients in the intensive care units, especially in mechanically ventilated subjects [65].

New synthetic opioids chemically unrelated to fentanyl have emerged on the global drugs market since 2010, contributing to the outspread of opioid crisis in US. Carfentanil is one of the most potent opioids, approved only for veterinary use as a general anesthetic agent for big animals. It has approximately 10,000 times higher potency than morphine and 100 times than fentanyl. The illicit use of this drug, mis-sold with other drugs, including heroin, led to hundreds of opioid overdoses, many of them being fatal [66].

#### Heroin

Heroin, also known as diacetylmorphine, is a highly addictive opioid drug which easily penetrates the blood–brain barrier and is rapidly converted to morphine in the brain. People using heroin typically report feeling of pleasurable sensation, called “rush,” due to the fast onset of euphoria after intravenous injection [67]. Heroin is prescribed as a strong pain medication in the UK, while it is not accepted in the US where it is more used as a drug of abuse. The high rate of abuse liability along with the rapid occurrence of physical withdrawal makes this drug a dangerous opiate linked to a huge number of overdoses [68, 69]. The raised danger of this drug led the Food and Drug Administration (FDA) to approve naloxone, for rapid overdose emergencies and methadone or buprenorphine, as a maintenance and replacement therapy in cases of heroin dependence [70].

#### Buprenorphine

Buprenorphine is a synthetic opioid derived from thebaine and approved by the FDA to treat acute and chronic pain and, in addition, in the maintenance and substitution treatment of opioid use disorder [71]. Buprenorphine is described as an atypical opiate. It acts at all opioid receptors with different affinity and activity. Indeed, it shows a potent *µ*-opioid receptor partial agonism and acts as antagonist with a high binding affinity at the DOR and KOR and as an agonist with lower binding affinity for NOP receptor [34]. Partial agonism at the *µ*-opioid receptor does not provide partial analgesia but instead analgesia similar to that of full *µ*-opioid receptor agonists as morphine or fentanyl [72]. These pharmacological properties allow a potent analgesia with less side effects and more safety advantages compared with full *µ*-opioid receptor agonists [72]. Moreover, the preferential spinal site of action rather than the brain may explain the better tolerability of this drug with less central effects such as euphoria and addiction [72, 73]. Interestingly, experimental results indicated that supraspinal injection of naloxone did not fully block the analgesic effects of buprenorphine [74], so suggesting an additional supraspinal component that might explain the unique preclinical and clinical profiles of this drug. However, hypotension, palpitation, tinnitus, QT prolongation, and upper respiratory infection have been reported as side effects [2].

As observed in mice, buprenorphine counteracts the antinociceptive and rewarding actions of morphine, raising the possibility that these effects of buprenorphine can also be affected by its ability to co-activate the NOP receptor [75]. Moreover, the development of tolerance in rodents treated with morphine is faster than in buprenorphine-treated rodents, assuming that activation of the NOP receptor may also contribute to the limited tolerance associated with buprenorphine [76]. Buprenorphine administration in rats produced fewer reward signs than classical opioid agonists administration, probably because of its ability to act at multiple targets [34].

#### Methadone

Methadone occurs in R-enantiomeric and S-enantiomeric forms, with R-methadone that presents a better pharmacological activity [77]. It is a synthetic opioid acting as a full agonist of *µ*-opioid receptor but also as NMDA receptor antagonist. The blockade of NMDA receptor can probably explain the lower rates of tolerance than other opioids like morphine, as demonstrated in rat models of neuropathic pain [78, 79]. This opiate is primarily used in heroin maintenance. Moreover, due to its multi-mechanistic pharmacological profile, it is an interesting drug to treat severe chronic pain often characterized by hyperalgesic states (e.g., cancer pain) and in opioid rotation protocols in patients experiencing inadequate pain relief or unbearable side effects with other opioids [2, 80–82]. Methadone exhibits similar side effects to morphine. Additionally, its use has been associated to a delayed respiratory depression, prolonged QT interval, and torsade de pointes.

#### Tapentadol

Tapentadol is a strong analgesic that shows a dual mechanism of action: indeed, the combination of μ-opioid receptor agonism and norepinephrine reuptake inhibition (NRI) generates a synergistic analgesic action [83]. Conversely to tramadol, which is clinically classified in the second step of the WHO analgesic ladder, tapentadol is a strong analgesic, with a reduced MOR load, due to the lower MOR binding affinity compared to morphine [84]. Moreover, tapentadol is not a prodrug and does not undergo CYP-mediated metabolism; its minimal serotoninergic activity accounts for limited serotoninergic adverse effects (nausea, vomiting, serotoninergic syndrome risk) [85]. Thus, tapentadol is well-tolerated among patients both for chronic cancer [86] and non-cancer pain [83, 87]. The NRI action seems relevant in the treatment of chronic neuropathic pain, as confirmed by studies using mice with a genetic deletion of MOR where tapentadol partially maintained its analgesic effects [88]. Moreover, in another preclinical study, tapentadol inhibited the spontaneous electrophysiological activity of locus coeruleus neurons, and this inhibitory effect was reversed by both α-2 receptor antagonists and MOR antagonists, suggesting a synergistic participation of these two receptors in pain modulation [89]. In addition, tapentadol shows an abuse liability lower than other *µ*-opioid receptor agonists, suggesting its good tolerability and safety profile [90, 91].

#### Codeine

Codeine is a naturally occurring opioid because of its presence in opium from the poppy plant. It is an agonist of *µ*-, *κ*-, and *δ*-opioid receptors even if it has more affinity for *µ* than the other opioid receptors [92]. Codeine is metabolized and activated by cytochrome P450 2D6 (CYP2D6) into morphine with 10 times higher potency than codeine. In patients leading multiple copies of this cytochrome, we can expect to react more than the 50% of conversion into morphine, with consequent high toxicity [93]. Codeine is used as cough sedative to relieve chronic cough and as a step 2 analgesic for the mild-moderate pain therapy in combination with acetaminophen or ibuprofen [94]. Conversely to morphine, codeine is not a P-glycoprotein substrate; therefore, its onset of action is faster than equianalgesic dose of morphine [95]. Similar to other opioid drugs, side effects such as respiratory depression and physical dependence can occur after chronic or excessive use, particularly in extensive metabolizers due to the faster and increased production of morphine through CYP metabolism.

#### Tramadol

Tramadol is an analogue of codeine with comparable analgesic effects. Similar to the other opiate herein described, tramadol shows affinity especially for *µ*-opioid receptors, although its binding affinity is lower than morphine [96]. In addition to the agonism for MOR, tramadol exerts its analgesic activity through the inhibition of norepinephrine and serotonin reuptake [92]. This compound consists of two enantiomers, both of which contribute to analgesic activity via different mechanisms: ( +)-tramadol inhibits serotonin reuptake, while ( −)-tramadol inhibits norepinephrine reuptake [97]. In the liver, tramadol is O-demethylated by cytochrome P450 (CYP2D6) to form the active metabolite O-desmethyltramadol, which exerts its full analgesic effect [98]. Preclinical studies in rats indicate that tramadol has a low abuse potential than classical opioids, suggesting that this mixed pharmacology of tramadol may limit these adverse effects [99]. Despite tramadol induces less severe side effects than morphine in terms of respiratory depression and gastrointestinal effects, the common side effects of the other opiates along with serotoninergic syndrome risk have been also reported upon its administration [92].

### Bifunctional agonists

Given the wide range of adverse effects described for MOR agonists, in recent years, research on analgesics focused on new drugs that possess, in addition to binding to the *µ* receptor, also affinity for other receptors. The strategy is based on the maintenance of good levels of analgesia, overcoming side effects. Mixed pharmacology deals with those drugs that possess affinity for different receptors. These drugs can be simultaneously agonist for two receptors (mixed agonist) or, moreover, agonist for one receptor and antagonist for another (mixed agonist–antagonist). In this section, we will review the promising mixed target profiles that bind at the same time different opioid receptors like *µ*-NOP agonist, *µ*-*κ* agonist, *µ* agonist-*δ* antagonist, *µ*-*δ* agonist, and *κ*-*δ* agonist.

#### µ/NOP agonists

The role of NOP receptors in analgesia is complex because it depends on the animal models implicated. Indeed, in rodents, it can provide analgesia, or not, depending on the route of administration [100]. For example, in mice, N/OFQ ligand could block the antinociceptive activity of *µ*-opioid agonists, owning no antinociception activity when administered intracerebroventricularly [101]. Instead, in nonhuman primates (NHPs), it exerts an antinociception activity supraspinally, spinally, and systemically [102]. However, the activation NOP receptor seems to be able to reduce some typical MOR agonists side effects including respiratory depression and physical dependence in both rodents and nonhuman primates [100].

*Cebranopadol* is a novel and promising molecule with dual *µ* and NOP agonist proposed to treat acute and chronic pain. As reported by clinical studies, cebranopadol showed long-lasting analgesia and shorter side effects in respect to morphine, thus suggesting its safer pharmacological profile [103]. Studies carried out in rodents reported that even at higher doses, cebranopadol showed reduced ability to produce physical dependence and respiratory depression as well as to influence motor coordination compared to morphine [104]. In addition, it exerts analgesic, antiallodynic, and antihyperalgesic properties in acute inflammatory pain as well as in neuropathic and cancer pain models [104]. Given these findings, cebranopadol represents a new opportunity for the treatment of several chronic pain states.

Moreover, particular interest in this field has been also devoted to other MOR/NOP bifunctional compounds such as *BU08028*, an analogue of buprenorphine, *BU10038*, and *AT-121*. Recent preclinical studies report that these molecules exert an analgesic effect similar to morphine with reduced abuse liability, respiratory depression, and acute physical dependence [34, 105–107].

#### µ-κ agonists

The activation of the *µ* receptor determines analgesia, however, along with a series of side effects such as respiratory depression, constipation, physical dependence, and reinforcing effects on CNS. On the contrary, *κ*-opioid receptor stimulation induces antinociception without respiratory depression and constipation. However, KOR stimulation has been associated with dysphoria [108]. *Dezocine* is a new µ-κ partial agonist showing antinociceptive properties with fewer adverse events and is currently approved in China for the treatment of moderate-to-severe pain [109]. Similarly, the morphine-derived *PPL-101* and *PPL-103* (partial agonists at *µ*-, *κ*-, and *δ*-opioid receptors) as well as *MP1207* and *MP1208*, which act as µ-κ partial agonists, have been reported to produce a potent antinociceptive activity without inducing conditioned place preference/aversion in mice [110, 111].

#### µ agonist-δ antagonists and µ-δ agonists

Another class of bifunctional molecules is represented by *µ* agonist-*δ* antagonists. The discovery of these ligands was based on the idea that the *δ* activation causes some of the negative side effects of *µ*-opioid agonists, as observed in studies in which *δ* antagonists blocked morphine-reinforcing behavior [108]. The major exponents of this drug family are represented by *mitragynine* and *SRI-39067* that as shown exhibit fewer abuse liability, reduce the reinforcing effects of morphine [34, 112], and promote less tolerance and withdrawal than morphine in rodents [113].

If the antagonism of *δ* receptors should lead to maintaining analgesia with less side effects, it is apparently strange that the dual agonists µ/δ benefit from the activation of the *δ* receptors with less side effects. In this regard, the peptide *biphalin* [114] and *SRI-22141* [115] showed a potent antinociceptive property along with reduced tolerance and dependence in mice with neuropathic pain. This evidence suggest that the analgesic usefulness produced by *δ*-receptor activation could occur only in pain conditions associated with an inflammatory state [108].

#### κ-δ agonists

Finally, there are some studies that reported an involvement of both *δ*- and *κ*-opioid receptors in modulating pain relief. One problem of these compounds is that *δ* receptors activation may cause convulsions, an important side effects that can be countered by *κ*-receptor activation. Among the compounds of this class, *MP1104* is the one of the most promising. Studies in mice reported that this compound showed antinociceptive effects [108] with less typical aversive side effects of *κ*-receptor activation.

### Biased agonists

The term “functional selectivity,” introduced in 1998, describes the idea that different agonists binding to specific residues at the orthosteric site of a GPCR can cause distinct conformational changes in the intracellular loops, resulting in different signaling outcomes.

This phenomenon, also known as “biased agonism,” indicates the ability of a specific ligand to preferentially activate one signaling pathway over another. In particular, the idea of functional selectivity suggests that imparting selectivity for one signaling pathway over another can provide a mean to separate beneficial effects from adverse effects of a drug.

The discovery of this phenomenon has garnered significant attention in the opioids field.

It is well known that activating opioid receptors can trigger the G-protein and/or *β*-arrestin2 transduction pathways. The G-protein pathway activation is responsible for the canonical signaling that induces opioid analgesia, while the *β*-arrestin pathway regulates opioid receptor signaling (including desensitization and internalization) and is generally associated with the emergence of opioid side effects. These findings [116, 117] laid the foundations for development of a series of promising MOR-biased agonists that will be briefly described in this section [16, 118–120].

*Oliceridine* (Olinvyk™, Trevena, Inc.) represents a novel G protein-selective MOR agonist approved in 2020 for use by US FDA for the treatment of moderate-to-severe acute pain management enough to require an intravenous opioid analgesic and for whom alternative treatments are inadequate [121, 122]. Its clinical use is currently being tested in Europe and Asia as well [123]. Despite oliceridine did not show structural similarities to morphine, this molecule acts as a potent partial agonist in G protein signaling and seems to produce little *β*-arrestin2 recruitment. In particular, this molecule preferentially activates the MOR receptor, resulting in decreased cAMP activity and producing analgesia. Conversely, oliceridine reduces MOR activation of *β*-arrestin, which is associated with the development of certain opioid-induced side effects, including respiratory depression and tolerance. Furthermore, it has been reported that oliceridine causes less receptor internalization and significantly lower phosphorylation compared to morphine [124, 125]. In vivo studies also showed that oliceridine was able to induce a fourfold more potent analgesic effect than morphine, and that differently from this latter oliceridine treatment leads less tolerance and OIH [126, 127]. However, similarly to other conventional opioids, it maintains an abuse potential [126, 127]. The improved pharmacological profile of this functionally selective opioid was confirmed in various clinical multicenter trials involving a wide range of patients. These trials demonstrated oliceridine’s ability to provide potent analgesic efficacy with an enhanced safety and tolerability profile [128–132]. In the recent years, research has also focused on other MOR biased ligands and in particular to *PZM21* [133, 134].

Studies in rodents and NHPs showed that PZM21 was able to promote a long-lasting dose-dependent antinociception with reduced respiratory depression [133, 135, 136]. However, some aspect regarding its ability to induce tolerance and abuse liability leads to ongoing debate about its safer profile in respect to conventional opiates [133, 136, 137].

Moreover, preclinical study highlighted the ability of substituted piperidine benzimidazoles including *SR-17018*, *SR-14968*, *SR-15098*, and *SR-15099*, to promote, similarly to conventional opioids (morphine and fentanyl), a long-lasting antinociception effects with less respiratory depression [138, 139].

However, further studies are needed to evaluate the future development and potential application of these molecules in humans.

Although most studies have primarily focused on MOR, also KOR-biased agonists (e.g., triazole 1.1, LOR17) have been recently proposed as promising candidates for treating itch and pain [140–142]. In this regard, growing evidence indicates that preferential activation of KOR-mediated G protein signaling over *β*-arrestin2 recruitment can lead to antinociceptive and antipruritic effects without inducing dysphoria and sedation in different animal models of chronic pain including inflammatory and neuropathic pain [140, 141, 143].

In addition, also peripherally restricted KOR agonists have been developed in order to avoid sedative and dysphoric side effects related to CNS KOR activation. This approach led to the development of different compounds, including CR845 [142]. This latter has been tested in several phase 2/3 clinical trials for postoperative analgesia and uremic pruritus [144, 145]. However, in 2021, it was approved in the United States for treating moderate-to-severe pruritus only, in patients undergoing hemodialysis [142].

## Conclusion

This review offers an overview of the endogenous opioid system, emphasizing the fundamental role of its modulation for ensuring effective pain relief. Indeed, the activation of opioid receptors is essential for producing both analgesia and side effects [27]. Although opioid use is linked to numerous adverse events, their rational use remains one of the most appropriate pharmacological approach for treating different, albeit not all, conditions of chronic pain.

The advancement of scientific research in this field will provide new analgesic molecules endowed with improved safety and tolerability profiles which could be useful to ensure better pain management.

## Data Availability

No datasets were generated or analysed during the current study.
